# A 21st Century Principle-Based Training Approach to Psychotherapy: A Contribution to the Momentum of Transtheoretical Work

**DOI:** 10.32872/cpe.11925

**Published:** 2024-04-26

**Authors:** Anna Babl, Catherine F. Eubanks, Marvin R. Goldfried

**Affiliations:** 1 Department of Clinical Psychology, Leiden University, Leiden, The Netherlands; 2 Department of Psychology, Stony Brook University, New York, NY, USA; Department of Psychology, University of Trier, Trier, Germany

**Keywords:** principles of change, psychotherapy training, Alliance-Focused Training, deliberate practice, markers

## Abstract

**Background:**

Despite the finding that the majority of psychotherapists adopt a rather process-oriented and integrative stance, it is uncommon that psychotherapy trainings are transtheoretical and transdiagnostic. Considering principles of change that cut across different schools of therapy holds promise for developing truly research-informed psychotherapy trainings. Common principles of change may answer the question what should be trained. Another important question is how to train. In current psychotherapy training programs, transfer of theory into practice relies mainly on role-playing exercises and supervised practice, both of which have their limitations.

**Aims:**

A fantasy for the future would be the development, implementation, and evaluation of a complementary 21st century online principle-based and marker-led psychotherapy training: incorporating the concepts of deliberate practice as well as expert training, the huge potential of technologies, and considering the importance of (context) responsiveness.

**Conclusion:**

To illustrate this idea, we present a training that we are currently developing, an online Alliance-Focused Training.

## Highlights

- Considering principles of change that cut across different schools of therapy holds promise for developing truly research-informed psychotherapy trainings.
- A fantasy for the future would be the development, implementation, and evaluation of a complementary 21st century online principle-based and marker-led psychotherapy training.
- We are currently developing, an online Alliance-Focused Training.

For a long period of time it was common for psychotherapists to work exclusively within their own theoretical framework – referred to by Norcross (2005) as an “ideological cold war.” Those first-generation approaches to psychotherapy, as developed by their founders, neglected or even suppressed and fought concepts and findings that were not in line with their original stance (Grawe & Caspar, 2011). Despite substantial theoretical and practical differences, and some evidence suggesting some advantages for certain approaches when treating certain conditions (e.g., Marcus et al., 2014), most comparison studies have found that bona fide therapies are equally effective (e.g., Wampold & Imel, 2015). As Saul Rosenzweig (1936) presciently observed when he quoted the Dodo bird from Lewis Carroll’s *Alice’s Adventures in Wonderland,* "everybody has won, and all must have prizes."

Given the lack of sizable differential treatment effects across psychotherapy orientations, increasingly more attention is being paid to the impact of the therapist on treatment outcome. Stiles and Horvath (2017) proposed that therapists’ responsiveness may be a key component of effective therapy: therapists are responsive by flexibly tailoring their relational connection and interventions to support individual patients’ needs in that moment (Stiles, 2009) in the context of their transdiagnostic characteristics (Hayes & Hofmann, 2020). Based on a qualitative meta-analytic review on therapist responsiveness, Wu and Levitt (2020) concluded that in order to be responsive, therapists need to develop awareness of and attunement to the process of therapy and to markers of shifts in patients’ experience.

Despite the finding that the majority of psychotherapists adopt a rather process-oriented and integrative stance (Norcross & Rogan, 2013), it is uncommon that psychotherapy trainings are transtheoretical and transdiagnostic; rather, the field remains entrenched in a training approach in which different theoretical orientations are siloed and implicitly pitted against one another. Given how much training is based on theoretically specific methods, it is surprising how little evidence there is for its effectiveness, especially comparing different theory-specific approaches (Knox & Hill, 2021).

## What to Train

One promising way to advance previous approaches to training is to consider principles of change that cut across different schools of therapy as complementary training modules when aiming for truly research-informed psychotherapy trainings. Transtheoretical and transdiagnostic change principles can help us identify and focus on areas of unity rather than develop new training approaches. In the following, a few key efforts to identify such principles of change are outlined.

An important landmark was Goldfried’s (1980) attempt to identify a set of change principles. He argued that change principles are located at an intermediate level of abstraction between the more abstract level of theoretical framework and the more concrete level of specific techniques. At this intermediate level of abstraction, it is possible to grant therapists some freedom with regard to the specific interventions they choose to apply with a specific patient in a specific situation but at the same time ensure that important change processes are facilitated. Drawing on the research literature, Goldfried (1980; Eubanks & Goldfried, 2019) proposed the following 5 principles of change shared across the major theoretical orientations:

- Fostering the patient’s hope, positive expectations, and motivation
- Facilitating the therapeutic alliance
- Increasing the patient’s awareness and insight (e.g., awareness of connections between thoughts, feelings, needs, actions)
- Encouraging corrective experiences (i.e., encouraging patients to take risks and engage in new behaviors that lead to a shift in cognitions and emotions)
- Emphasizing ongoing reality testing (i.e., helping patients to process corrective experiences and consolidate positive changes by recalibrating their expectations and self-views to be in line with their new reality)

Klaus Grawe and colleagues were also interested in change processes in psychotherapy, with the aim of developing a research-informed psychotherapy that would flexibly use all empirically supported mechanisms of change in psychotherapy (Caspar & grosse Holtforth, 2010). Based on a meta-analysis of approximately 900 comparative outcome studies on the effectiveness of psychotherapy (Grawe et al., 1994), they identified five general change factors:

- Problem mastery/coping (i.e., the patient learns to cope with difficult or anxiety-provoking situations)
- Clarification of meaning (i.e., the patient gains greater understanding of the source of their difficulties)
- Problem actuation (i.e., the patient’s emotional experience of the problem is activated during psychotherapy, to provide the optimal opportunity to foster change)
- Resource activation (i.e., the patient’s own resources—motivation, skills, strengths—are activated in the service of change)
- Therapeutic relationship

Another effort to identify principles of change with empirical support is the work by Castonguay and Beutler (2006). The five categories of principles they identified are framed in terms of guiding therapists as they predict how therapy will go and determine how best to intervene:

- Patient prognostic principles (i.e., patient characteristics that predict good treatment outcome such as baseline impairment, personality disorder, attachment, expectations, stage of change)
- Treatment/provider moderating principles (i.e., patient characteristics, often present at baseline, that therapists should be responsive to such as patient resistance, ambivalence, coping style)
- Patient process principles (i.e., patient during-treatment behaviors that facilitate or interfere with improvement such as active participation or resistance, respectively)
- Therapy relationship principles (i.e., elements of the patient-therapist exchange that facilitate or interfere with improvement such as alliance quality, alliance rupture repair, therapist empathy, therapist positive regard)
- Therapist intervention principles (i.e., therapist during-treatment behaviors that either facilitate or interfere with improvement such as receiving feedback based on routine outcome monitoring, being flexible, fostering more emotional experiencing and behavior change)

The different attempts to identify transtheoretical principles of change all have agreed on the therapeutic relationship or alliance as a key component of effective therapy. Many original studies and meta-analyses positioned the therapeutic alliance as a robust predictor of psychotherapy outcome across a wide range of patient diagnoses and different treatment types (e.g., Flückiger et al., 2018, 2020). This is true for both between-patient alliance effects (Flückiger et al., 2018) and within-patient early alliance effects on posttreatment outcome (Flückiger et al., 2020). Further, a survey of a diverse pool of 1,998 psychotherapy clinicians on the perceived presence of the five principles of change identified by Goldfried indicated strongest consensus for the therapeutic alliance and when participants estimated whether the principles were common to all schools of therapy, strong consensus was only indicated for the therapeutic alliance (Twomey, O’Reilly, & Goldfried, 2023).

## How to Train

Common principles of change may answer the question about what should be trained, but another important question is how change principles can be implemented in psychotherapy training. Research has found mixed evidence as to whether standard methods of therapist training are effective (Perlman et al., 2020). In current psychotherapy training programs, transfer of theory into practice with actual patients relies mainly on role-playing exercises and supervised practice. However, both forms of learning have their limitations: role-playing exercises might not be realistic and may thus fail to provide the trainee with useful preparation (Beutler & Harwood, 2004), while supervision usually only follows the trainee’s contact with the patient with some time-delay. Immediate feedback, for example in the context of live supervision, can only be realized at high cost and applied to a sub-sample of patients and relevant clinical situations. No satisfactory training procedure is currently in place by which novice psychotherapists can obtain in-vivo hands-on experience in dealing with the range of problems presented by a variety of patients, and in which they get immediate, accurate and consistent feedback (Beutler & Harwood, 2004). For a long time, the field of psychotherapy research has lacked a successful model for therapist skill advancement (Rousmaniere et al., 2017).

The study of expertise in other fields provides a potential model for understanding the key mediating factors involved in the development of top-level performers in psychotherapy. Across a variety of domains, researchers have found that engagement in extended, deliberate practice facilitates incremental development, resulting in superior performance (Chow et al., 2015). According to Ericsson (2006), deliberate practice is defined as individualized training activity especially designed to improve specific aspects of an individual’s performance through repetition and successive refinement. Empirical research suggests that deliberate practice can significantly improve the effectiveness and efficiency of psychotherapy education and training (e.g., Rousmaniere et al., 2017). Bailey and Ogles (2023) go as far as making deliberate practice suggestions for rupture and repair interventions, such as video-assisted observation of your work, getting consultant feedback, setting small incremental goals, solo deliberate practice, and feedback-informed treatment. An important next step would be its online application, with the advantages of easy and flexible availability of the training and its time-independent use (Berger, 2015). A meta-analysis of 201 studies has shown that in the health professions, internet-based learning was associated with large positive effects compared with no intervention and with equal effects compared to non-internet instructional methods (Cook et al., 2008). A systematic review synthesized the mental-health training literature published since 2010 to evaluate how different training models affect therapists’ knowledge, beliefs, and behaviors (Frank, Becker-Haimes, & Kendall, 2020). With regard to online training (20 studies), there was clear evidence that it can improve therapist knowledge, skills, and use of the intervention after online training (Frank et al., 2020).

One promising framework for guiding therapist responsiveness that has been proposed by Constantino and colleagues (2013) is context-responsive psychotherapy integration (CRPI), a transdiagnostic if-then approach. Based on empirical associations with therapy outcomes, Constantino and colleagues identified several patient characteristics and treatment processes that therapists will encounter and to which they need to react and be responsive, including low outcome expectations, ambivalence/resistance, patient self-strivings, alliance ruptures, and alarm signals from outcome monitoring (Constantino et al., 2013). Trainees can be taught to recognize markers of these common characteristics and processes and to select from several principle-driven, evidence-based methods to address the markers (*if* this occurs, *then* try one of these responses). CRPI is a promising model that requires more empirical support and further investigation of commonly occurring markers (Constantino et al., 2017). Focusing on *markers* as indicators of problems as well as patients’ readiness to work on those problems is a defining feature of Alliance-Focused Training (AFT; Muran & Eubanks, 2020). AFT works with markers of ruptures in the alliance during the psychotherapeutic process as indicators that it is time to pay close attention to the therapeutic relationship and be curious about what is taking place.

## A New Vision for Psychotherapy Training

Combining the questions “what to train” and “how to train”, the first and second author of this article are developing an online AFT as a concrete example and starting point for our 21^st^ century online principle-based and marker-led psychotherapy training. The online AFT will be modeled after the AFT approach to training and supervision developed by Muran, Safran, and Eubanks, which aims at helping therapists recognize and negotiate ruptures in the therapeutic alliance both through observation of patient and therapist behaviors, as well as attending to the therapists’ own internal emotional experience (Muran & Eubanks, 2020). The effects of AFT have been shown to foster rupture repair and patient outcome in six studies (Eubanks et al., 2019), including a randomized controlled trial (Muran et al., 2018).

In psychotherapy research, the alliance is typically conceptualized as consisting of the patient-therapist affective bond, and a purposeful collaboration on the tasks and goals of therapy (Bordin, 1979). It has been shown that during treatment, the alliance is characterized by rupture-repair episodes (e.g., Eubanks, Muran, et al., 2018). Ruptures are defined as moments of weakness or deterioration in the alliance (Eubanks et al., 2015) and they can be organized into two general categories: *confrontation ruptures*, in which patients or therapists move against the other person or the work of therapy, typically showing their concern directly; and *withdrawal ruptures,* in which patients or therapists move away from the other person or the work of therapy, usually having difficulties either recognizing their feelings or directly expressing them. The following three markers are indicators of confrontation ruptures: complaining/criticizing, pushing back, and controlling/pressuring (Eubanks & Muran, 2022); these three markers suggest the occurrence of withdrawal ruptures: shutting down, avoiding, and masking one’s own experience (Eubanks & Muran, 2022). Ruptures are common events (Muran & Safran, 2016), which is why it is important that there is a chance to repair them by means of resolution strategies (Eubanks, Muran, et al., 2018). Resolution strategies can include immediate strategies, in which the alliance rupture is immediately addressed and then the dyad returns to the therapy task they were previously engaged in. Examples of immediate strategies include changing the task or goal, illustrating the task or providing a rationale, and redirecting or refocusing on a therapy task (Eubanks & Muran, 2022). Ruptures can also be addressed using expressive repair strategies, which involve exploring the rupture in depth and can include inviting the other to explore the rupture, validating their experience of the rupture, and disclosing one’s own experience of the rupture. In addition, therapists and patients can address ruptures by acknowledging their own contribution to the rupture and by linking the rupture to larger interpersonal patterns in the patient’s life.

Online AFT will begin with short, introductory theoretical videos (based on the research evidence on rupture and repair) as well as videos of prototypical case examples of confrontation markers and withdrawal markers, which indicate potential ruptures in the alliance, as well as corresponding immediate and expressive resolution strategies. In a second part of the training, therapists will be provided with various patient-therapist video scenarios and will be encouraged to recognize markers of alliance ruptures when they occur as well as corresponding rupture resolution strategies. Exercises will increase in difficulty as therapists move from recognition (e.g., selecting options from a predefined list) to recall. They will receive immediate computer-generated feedback and the possibility to reflect on and refine their responses. Therapists will then practice their skills in recognizing and negotiating alliance ruptures by responding to short video scenarios of withdrawn, confrontational or otherwise interpersonally complex patients. Their responses as therapists will be video recorded and played back to them accompanied by questions designed to foster their curiosity about the therapeutic process as well as access to their own internal experience. Thereby, the online AFT implements deliberate practice for rupture and repair interventions as previously suggested (Bailey & Ogles, 2023). An essential aspect of developing online AFT will be collecting data on its efficacy and based on that data, refining and adapting the training as needed to ensure that it meets the aim of improving therapist skills and treatment outcomes.

A fantasy for the future would be the development, implementation, and testing of additional modules of a *21^st^ century online principle-based and marker-led psychotherapy training*: incorporating the concepts of deliberate practice as well as expert training, the huge potential of technologies, and considering the importance of (context) responsiveness. Such a training could include an online library of a range of clinical markers (e.g., a patient showing vulnerability may best be met with empathy on the side of the therapist. A bodily felt sense or abstract, intellectualized talk about emotions may be encountered with focusing interventions to enable emotional deepening. Self-critical processes may be played out using two-chair dialogue and uncompleted processes with significant others may be attended to by means of empty-chair dialogue to increase self-compassion and acceptance of emotions and needs) with example patient-therapist videos of how to respond to them (thereby already building a bridge to practice) together with a brief description of relevant basic and applied research. Markers are nested within and can be classified under change principles so that relevant ones can be searched for when needed. In a second step, therapists could be provided with videos of clinical scenarios and corresponding exercises of increasing degree of difficulty (e.g., answers to choose from are given, answers must be generated and written down to meet certain keywords, therapists receive immediate and automated feedback on their choices with the possibility to repeatedly refine their responses, therapists immediately respond to patient scenarios and their responses are video-recorded, the recordings are played back to the therapists together with questions facilitating self-observation and self-reflection, they are then invited to respond again and differently to the same patient scenario). The idea is that therapists can practice their skills in a safe and supportive environment before applying them to their work with actual patients. Such a training could be distributed easily and inexpensively, offers time-independent use, and provides a cost-effective model for therapist skill advancement and ultimately psychotherapy success at the individual patient level. It could complement traditional psychotherapy trainings of different approaches: it could be used by professors in classes, as well as by supervisors who could access resources from the online library while meeting with a trainee in individual or group supervision. Such a training could also facilitate lifelong learning: it could be incorporated into continuing education programs as well as being accessed by practicing therapists when they want to review or learn new skills.

We would like to conclude with some broader ideas for the future of what we are suggesting as a principle-based and marker-led psychotherapy training. To really advance our understanding of how best to practice therapy and train therapists, we need to delineate a full range of principles of change, and we should regard “what works” as an empirical question. One important future direction for principles of change would be to see other principles and common factors enjoy the “success” that the therapeutic alliance has achieved in attracting the interest of researchers, practitioners, and trainers (Eubanks & Babl, in press). As more principles of change are in a position to receive that kind of attention, it will pave the way for more research looking at interactions between them (Norcross & Lambert, 2019). Given that principles of change cut across different approaches to therapy, many practicing clinicians have experience with them and can make valuable contributions by sharing how they understand and employ them. Researchers and clinicians can actively partner with each other in an effort to build an online 21^st^ century principle-based and marker-led psychotherapy training.
